# Period1 mediates rhythmic metabolism of toxins by interacting with CYP2E1

**DOI:** 10.1038/s41419-020-03343-7

**Published:** 2021-01-12

**Authors:** Wenhao Ge, Tao Wang, Yang Zhao, Yunxia Yang, Qi Sun, Xiao Yang, Yan Gao, Xi Xu, Jianfa Zhang

**Affiliations:** 1 Center for Molecular Metabolism, Nanjing University of Science & Technology, Nanjing, China; 2 Cambridge Suda Genome Resource Center, Soochow University, Suzhou, China

**Keywords:** Biocatalysis, Transcription

## Abstract

The biological clock is an endogenous biological timing system, which controls metabolic functions in almost all organs. Nutrient metabolism, substrate processing, and detoxification are circadian controlled in livers. However, how the clock genes respond to toxins and influence toxicity keeps unclear. We identified the clock gene *Per1* was specifically elevated in mice exposed to toxins such as carbon tetrachloride (CCl_4_). Mice lacking *Per1* slowed down the metabolic rate of toxins including CCl_4_, capsaicin, and acetaminophen, exhibiting relatively more residues in the plasma. Liver injury and fibrosis induced by acute and chronic CCl_4_ exposure were markedly alleviated in *Per1*-deficient mice. These processes involved the binding of PER1 protein and hepatocyte nuclear factor-1alpha (HNF-1α), which enhances the recruitment of HNF-1α to cytochrome P450 2E1 (*Cyp2e1*) promoter and increases *Cyp2e1* expression, thereby promoting metabolism for toxins in the livers. These results indicate that PER1 mediates the metabolism of toxins and appropriate suppression of *Per1* response is a potential therapeutic target for toxin-induced hepatotoxicity.

## Introduction

Most physiological processes in mammals display circadian rhythms that are driven by the endogenous circadian clock. Both central and peripheral clocks are operated by positive- and negative-feedback loops of circadian genes, such as *Clock*, *Bmal1*, *Period1 (Per1)*, *Period2 (Per2)*, *Cryptochromes1 (Cry1) and Cryptochromes2 (Cry2)*, *Rev-Erb-α*, and *Rora*. In the negative-feedback loops of circadian genes, the PER and CRY form complexes and shut down transcription initiated by CLOCK–BMAL1 heterodimers^1,2^. Circadian rhythms play an important role in maintaining homeostasis and solid organ function. Although disrupted rhythms can lead to metabolic impairments, the reverse is also true that disrupting metabolism can alter circadian rhythms^3^. Clock dysfunction accelerates the development of liver diseases such as fatty liver diseases, cirrhosis, hepatitis, and liver cancer, and these disorders also disrupt clock function^4^. On the other hand, patients with cirrhosis have liver portal hypertension and dysfunctional circadian clock systems^5^. Hepatic fibrosis induced by carbon tetrachloride (CCl_4_) in mice leads to alterations in the circadian rhythms of hepatic clock genes^6^. The overarching evidence that circadian homeostasis is critical to human health, and conversely, that some abnormal metabolism and diseases induce abnormal responses of the circadian clock and negatively affect health, cannot be ignored.

Acute liver injury induced by hepatotoxins has been recognized as one of the most important pharmacovigilance concerns and the leading cause of drug withdrawal on safety grounds^7,8^. After acute liver injury, parenchymal cells regenerate and replace the necrotic or apoptotic cells. The wound-healing response of the liver to repeated injury leads to liver fibrosis. Advanced liver fibrosis results in cirrhosis, liver failure, and portal hypertension and often requires liver transplantation^9^. The generation of reactive oxygen species (ROS) and oxidative stress resulted from the metabolism of hepatotoxins is a common mechanism of liver injury. Of the multiple forms of P450s present in the liver endoplasmic reticulum, cytochrome P450 2E1 (CYP2E1) has been implicated as a key metabolizing enzyme for various xenobiotics from food and drugs into toxic metabolites which lead to liver injury. Induction of *Cyp2e*1 with ethanol, pyrazole, or other chemicals has been shown to promote oxidative stress^10,11^.

Although endogenous circadian controlled daily metabolism of toxins and hepatic *Cyp2e1* expression have been described in mammals^12,13^, it still was unclear how the clock genes respond toxins, and how these responses influence the capacity to metabolize toxins. In present studies, we identified the clock gene *Per1* was specifically elevated in mice exposed to toxins such as CCl_4_. We demonstrated the PER1 regulated *Cyp2e1* expression, thereby promoting metabolism for toxins in the livers. These results suggest that PER1 is a regulator of liver metabolism of toxins. Appropriate suppression of the *Per1* response is a potential therapeutic target for toxin-induced hepatotoxicity and dysfunction of the circadian clock.

## Materials and methods

### Animals and treatment

Male, 8–10-week-old *Per1*^−/−^ and WT C57BL/6 mice were used in this study. The *Per1*^−/−^ mice used in this study have been characterized previously^14^. All mice were maintained under standard laboratory conditions, with 12-h light/12-h dark cycles and free access to food and water at all stages of the experiments. All procedures were approved by the Animal Care and Use Committee at Nanjing University of Science and Technology (ACUC-NUST-20160016).

A single intraperitoneal injection of CCl_4_ (0.6 mL/kg body weight) was administrated to WT and *Per1*^−/−^ mice. As controls, animals received the same volume of olive oil intraperitoneally. To determine the statistical significance of any observed differences, we used five male mice per time point following CCl_4_ administration, which included 24, 48, and 72 h. To detect the circadian mRNA expression of clock genes in response to CCl_4_, mice were killed at zeitgeber time (ZT)1 (ZT0 corresponds lights on and ZT12 to light off), ZT5, ZT9, ZT13, ZT17, ZT21, ZT24 (*n* = 5, each time point).To determine the plasma pharmacokinetics of CCl_4_, the blood samples were obtained from five male mice per time point following CCl_4_ administration, which included 2, 4, 8, and 12 h. For induction of liver fibrosis, CCl_4_ was injected twice weekly for 4 weeks. Mice were killed 48 h after the last injection. Serum aspartate transaminase (AST) and alanine transaminase (ALT) activity were measured using an AU2700 automatic biochemical analyzer (Olympus, Tokyo, Japan).

To determine the plasma pharmacokinetics of capsaicin, capsaicin was each separately suspended in 0.5% (w/v) CMC–Na solution to obtain a final concentration of 30 mg/ml. WT and *Per1*^−/−^ mice were given capsaicin at a dose of 300 mg/kg, via gavage. After oral administration, the blood samples (0.6 mL) were collected at the desired times (30 min, 1, and 3 h) into heparinized centrifuge tubes.

To determine the plasma pharmacokinetics of acetaminophen (APAP), APAP was dissolved in warm saline and injected intraperitoneally at the dose of 500 mg/kg body weight, whereas saline was administered to control animals. After 0.5, 1, 2, or 4 h. Blood samples from six different mice per time point were then quickly centrifuged at 4 °C and a small plasma volume from each mouse was rapidly stored to assess the concentrations of APAP.

### Quantitation of CCl_4_ by the headspace gas chromatographic method

Samples were prepared according to the method described by Jerry et al.^15^. Stoppered test tubes containing samples for CCl_4_ analysis were incubated at 60 °C for 15 min. All experiments were carried out by using Bruker GC-450 equipped with an electron capture detector (Bruker, Columbia, MD, USA). Column was 30 m × 0.25 mm i.d. capillary coated with 0.50 mm of 50% phenyl–50% methyl polysiloxane (Rxi-50). The chromatographic conditions were: column temperature, 100 °C: detector temperature, 200°C. Nitrogen was used as carrier gas.

### Determination of hepatic oxidative stress and GSH levels

ROS was measured using 2’, 7’-dichlorofluorescein diacetate as a probe. Lipid peroxidation was determined by measuring the formation of the thiobarbituric acid-reactive substances spectrophotometrically and was expressed as malondialdehyde (MDA) concentration. Hepatic glutathione (GSH) levels were estimated by a colorimetric method using Ellman’s reagent and glutathione reductase. Hepatic ROS, MDA, and GSH contents were assayed with commercial kits according to the manufacturers’ instructions (Jiancheng, Nanjing, China).

### TUNEL staining

At sacrifice, tissues were rapidly isolated, fixed in 4% paraformaldehyde, cryoprotected with 30% sucrose, and embedded in the optimal cutting temperature compound. The specimens were snap-frozen and sectioned into 15-μm sagittal sections (CM1950; Leica, Germany). Terminal deoxynucleotidyl transferase dUTP nick end labeling (TUNEL) staining was performed using a TUNEL Apoptosis Assay Kit (Beyotime, C1088). Nuclei were stained with 4′,6-diamidino-2-phenylindole (DAPI). Fluorescence images of liver slices were observed with fluorescence microscopy (Eclipse 800; Nikon, Tokyo, Japan).

### Flow cytometry

After treatment, cells were stained with annexin V and propidium iodide (PI) (Annexin V—early apoptosis detection kit, Beyotime Biotechnology) following the manufacturer’s instructions. In brief, after culture under various conditions, cells were harvested and suspended in the appropriate binding buffer, stained with fluorescein isothiocyanate-conjugated annexin V and propidium iodide at room temperature for 15 minutes, and subsequently analyzed by a FACSCalibur flow cytometer (BD Biosciences, San Diego, CA, USA). Cells positive for annexin V and annexin V/propidium iodide were considered early and late apoptotic cells, respectively. All cells negative for annexin V were considered viable cells.

### Histological analysis

Liver tissue was fixed in 10% phosphate-buffered formalin overnight, embedded in paraffin, and cut into 4 μm sections. Sections were stained with hematoxylin and eosin for routine examination or Masson’s trichrome for visualization of hepatic collagen deposition. Immunohistochemical staining was performed according to standard procedures^16^ using an appropriate specific primary antibody (Anti-CYP2E1, Abcam, Cambridge, UK; Anti-α-SMA, Millipore, Billerica, MA).

### Cells and treatment

HepG2 E47 cells expressing *Cyp2e1* (Boster Biological Technology Ltd., Wuhan, China) were cultured at 37 °C under 5% CO_2_ humidified atmosphere, using DMEM, supplemented with 10% FCS, 100 U/ml penicillin, and 100 mg/ml streptomycin.

Primary hepatocytes were isolated from 6 to 8-week-old male C57BL/6 mice using an in situ liver perfusion approach^17^. In brief, hepatocytes were dissociated from anesthetized adult mice by non-recirculating collagenase perfusion (C5138, Sigma, USA) through the portal vein. The isolated cells are then filtered through a 100 μm pore size mesh nylon filter. Cells were plated in collagen I–coated 6- or 12-well plated (at two or one million cells per well, respectively) in M199 medium plus 10% fetal bovine serum plus penicillin/streptomycin. After 3 hours of attachment, the medium was replaced with the appropriate assay medium.

For *Per1* knockdown experiments, the complementary oligonucleotide of small hairpin RNA targeting the 5′‐GGTGCTCCCTAACTATCTATT‐3′ sequence was chemically synthesized, subcloned into the lentiviral vector (GenePharma, Shanghai, China), and transfected into primary hepatocytes or HepG2 E47 cells. Cells were infected with lentivirus at low multiplicity according to the manufacturer’s instructions. Cells were selected in puromycin (1 μg/mL) and polyclonal populations were expanded and analyzed. A hairpin siRNA with no sequence homology to human genes provided by the manufacturer (GenePharma) was used as the negative control. *Per1* mRNA knockdown was assessed by real-time PCR. Clones that had sufficient knockdown (>75%) were used for further experiments.

For overexpression of human *Per1*, cells were transfected with pCMV-Sport2 Per1 or pCMV-Sport2 vector as control using Lipofectamine 2000 Transfection Reagent (Invitrogen, Carlsbad, CA). Construction of pCMV-Sport2 Per1 was characterized previously by Zheng and colleagues^18^.

The transfected cells were treated with 0.5% (v/v) CCl_4_ in 0.25% dimethyl sulfoxide prepared in serum-free culture medium for 24 h. All cell lines have been authenticated at the beginning of the study and again within 4 months after completion of the experiments. All cell lines have been tested for mycoplasma contamination frequently. Determination of MDA and GSH levels in cells were performed using commercial kits according to the manufacturers’ instructions (Jiancheng, Nanjing, China).

### RNA extraction and quantitative real-time PCR

Total RNA was extracted from samples with Karrol reagent (Karroten Scientific, Nanjing, China) according to the manufacturer’s instructions. Reverse transcript reaction was carried out by reverse transcript kit (Invitrogen, Carlsbad, CA) according to the manufacturer’s protocol. Real-time PCR was performed with the SYBR Green PCR Kit (Applied Biosystems, Foster City, CA) following the manufacturer’s instructions on an ABI 7300 real-time PCR system (Applied Biosystems) in a 20-μl volume. For an internal standard control, the expression level of glyceraldehyde-3-phosphate dehydrogenase (*Gapdh*) was simultaneously quantified. All primer sequences used for real-time PCR are shown in Table S1.

### RNA-sequencing for detection of differentially expressed genes and pathways

RNA-sequencing (RNA-Seq) analysis and quantification were utilized to investigate changes in liver mRNA profiles among the different treatments performed. Isolated RNA was sent to BGI Co., Ltd. for conducting RNA-seq, which was performed on a BGISEQ-500 (Shenzhen, China). All samples were replicated three times for confirmation purposes. All the generated raw sequencing reads were filtered to remove reads with adapters, reads in which unknown bases are >5%, and low quality reads. Clean reads were then obtained and stored as FASTQ format. HISAT^19^ was used to map clean reads to the genome of GRCm38.p6. NOISeq^20^ method was used to screen deferentially expressed genes (DEGs). All DEGs were mapped to terms in Kyoto Encyclopedia of Genes and Genomes (KEGG) pathway enrichment analysis.

### Western blot analysis

Proteins were extracted following the procedure described previously^21^. The extraction was separated by sodium dodecyl sulfate polyacrylamide gel electrophoresis (SDS–PAGE) 8–12% polyacrylamide gel and then electrically transferred to a polyvinylidene difluoride membrane. After blocking with 5% (w/v) BSA in TBST at room temperature for 1 h, the membranes were then incubated with an appropriate specific primary antibody (Anti-CYP2E1, Abcam, ab28146; anti-PER1, Abcam, ab3443; anti-HNF-1α, Cell Signaling Technology, #89670; anti-CBP, Cell Signaling Technology, #7389; anti-β-ACTIN, Bioworld, AP0060) at 4 °C overnight, followed by incubation with HRP-conjugated secondary antibody (Boster Biological Technology Ltd., BA1054) and detected by enhanced chemical luminescence kit (Thermo scientific, Hudson, NH, USA).

### Immunoprecipitation assays

Immunoprecipitation assays were performed as described previously^22,23^ with slight modification. For ChIP assays, cross-linked chromatin was immunoprecipitated with 5 μg of antibody (anti-HNF-1α, Cell Signaling Technology, #89670; anti-PER1, Abcam, ab3443), or negative control rabbit IgG (Beyotime, A7016) at 4 °C overnight. Immunoprecipitated DNA was then used as a template for PCR. All primer sequences used for ChIP-PCRs were listed in Table S1.

For co-immunoprecipitations, liver tissues were homogenized and lysed with a Non-denaturing lysis buffer containing 20 mM Tris-HCl pH 8.0, 137 mM NaCl, 2 mM ethylenediaminetetraacetic acid (EDTA), and 1% NP-40 with protease inhibitor cocktail (Boster Biological Technology). To prepare immunoprecipitates, we incubated lysates with antibody (anti-HNF-1α, Cell Signaling Technology, #89670; anti-PER1, Abcam, ab3443) overnight at 4 °C, and then incubated with Protein A-Sepharose 4B (Invitrogen). Immunoprecipitates were washed five times with wash buffer containing 10 mM Tris-HCl pH 7.4, 150 mM NaCl, 1 mM EGTA, 1 mM EDTA, 1% Triton X-100, 0.2 mM sodium orthovanadate with protease inhibitor cocktail, boiled in SDS–PAGE loading buffer. Proteins were analyzed by western blotting as described above.

### Quantitation of capsaicin by HPLC

Samples were prepared according to the method described by Yingying Zhao et al.^24^. A 200 μL aliquot of plasma was mixed with 50 μL α-naphthol (10 μg/mL; Aladdin, China), 10 μL methanol, 400 μL water, and 500 μL acetonitrile, and vortexing for 1 min. The solution was then extracted with 1.5 mL each of acetic ether and cyclohexane for capsaicin, followed by vortexing for 3 min. After 10 min centrifugation at 3000 rpm, 2.5 ml of the supernatant was transferred to another container and evaporated to dryness via nitrogen at 37 °C. The residue was dissolved in 300 μL of acetonitrile and mixed for 1 min. Following centrifugation at 3000 rpm for 5 min, 50 μL of the supernatant was injected into the High-performance liquid chromatography (HPLC) system for analysis.

Samples were analyzed using an HPLC (Waters 1525 System; Millipore, Bedford, MA, USA) on a reversed-phase C18 column. The mobile phase was acetonitrile–water (43:57) at a flow rate of 1.0 mL/min. The elution of metabolites was monitored at a wavelength of 254 nm.

### Quantitation of APAP by HPLC

Samples were prepared according to a method described previously^25^. A 100 μg/ml methanol solution of theophylline was added to the plasma at a ratio of 1:1. This mixture was allowed to precipitate for at least 10 min and centrifuged twice at 11,000 × *g* 6–7 min to pellet the precipitated proteins. The mixtures were filtered through 0.45-μm HV Millipore cellulose filters and 20-μ1 samples were injected immediately into the chromatographic system.

Samples were analyzed using an HPLC (Waters 1525 System; Millipore, Bedford, MA, USA) on a reversed-phase C18 column. The gradient elution started with 30% methanol passing through the column at a flow-rate of 1.5 ml/min. After a delay of 0.5 min the methanol concentration was increased linearly to 75% over 7.5 min. The column was returned to 30% methanol after a delay of 1 min. The variable-wavelength detector was set at 254 nm.

### Statistics

The single cosinor method was used for analysis of circadian rhythm^26^, and the cosine function equation was as follows: *Y* (t) = M + Acos (xt + u). The rhythm characteristics estimated by this method included the mesor (middle value of the fitted cosine representing a rhythm-adjusted mean), the amplitude (half the difference between the minimum and maximum of the fitted cosine function), and the acrophase (time of peak value in the fitted cosine function). Data were presented as means ± S.E.M. Statistical analysis was performed by Student’s *t* test, one-way ANOVA or two-way ANOVA followed by Tukey’s post hoc test. Significance was defined as *P* value < 0.05. Sample sizes of all experiments were predetermined by calculations derived from our experience. No sample was excluded from the analyses. Animals were not randomly assigned during collection, but the strain, sex, and age of the mice were the same, and the data analysis was single masked. Investigators were not blinded to the group allocation during the experiment and outcome assessment. The number of replicates was indicated in each figure legend. All qPCR data represented the mean of three technical replicates. The mean of the technical replicates was used per biological replicate. All statistical tests justified as appropriate and the data met the assumptions of the tests. There was an estimate of variation within each group of data.

## Results

### RNA sequence analysis of gene expression in the liver of mice exposed to CCl_4_

**Fig. 1:**
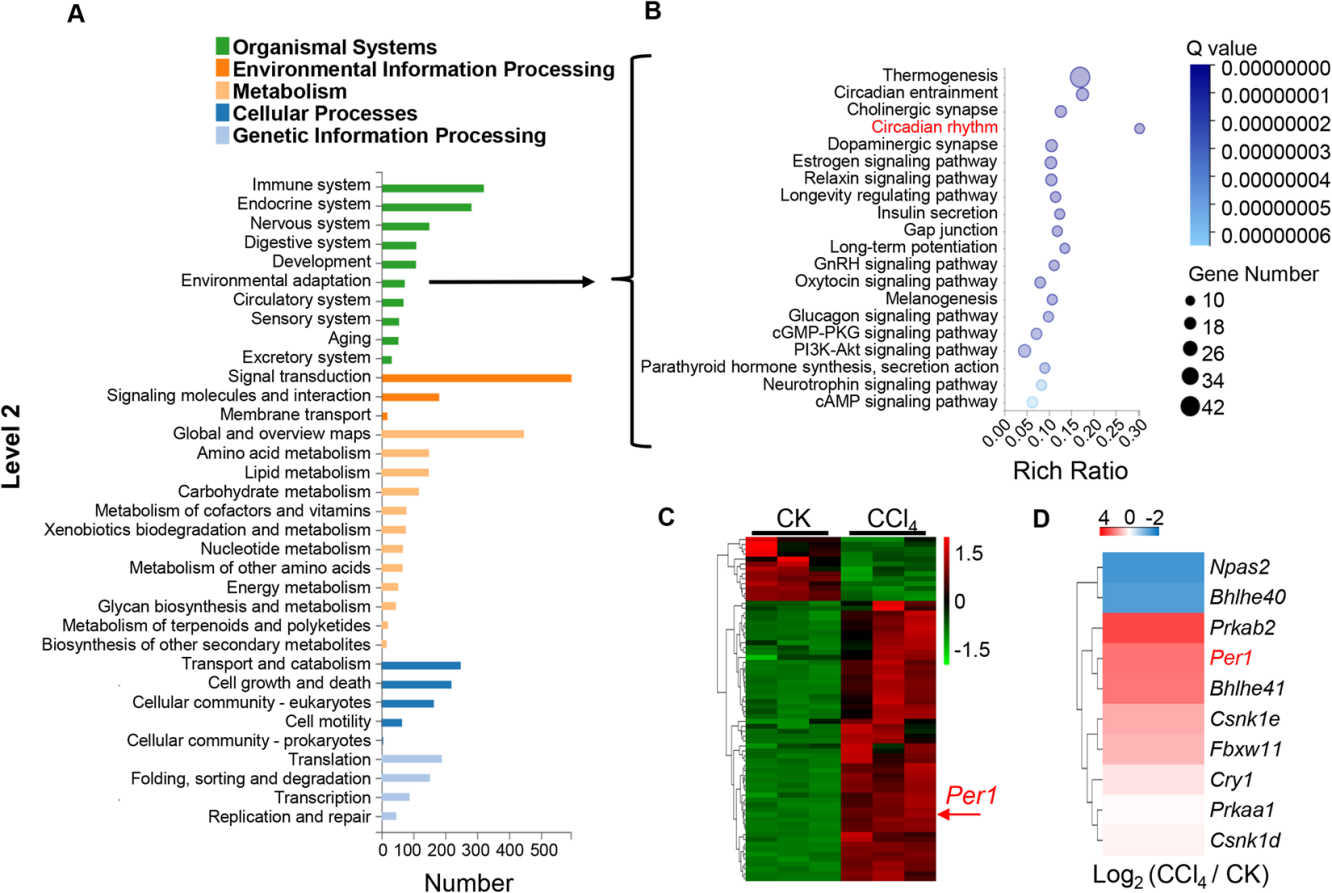
*Per1* mRNA levels were upregulated significantly in mice exposed to carbon tetrachloride identified by RNA-Seq analysis. Liver tissues were collected 24 h after a single CCl_4_ treatment. Samples were analyzed by RNA-seq and differentially expressed pathways. **A** Distribution of the level 2 KEGG pathways in both CK and CCl_4_. The bar chart showed the numbers of sequences that were assigned within different pathway categories. **B** Top 20 pathways in the environmental adaptation from **A** were shown. **C** Heat map of 70 DEGs in the environmental adaptation pathway from **A**. **D** DEGs in the Circadian rhythm pathway from **B** were shown. CK: control. *N* = 3 independent biological replicates per group.

To search possible response genes following toxins exposure, we performed RNA-Seq to analyze the hepatic gene expression changes between toxin CCl_4_-treated and oil-controlled mice. A KEGG pathway analysis showed that organismal systems pathways (KEGG level 1) were the major pathways altered in CCl_4_-treated liver and 2048 DEGs are involved in multiple pathways essential for CCl_4_-induced liver injury and fibrosis (Fig. 1A), and 70 DEGs in the environmental adaptation pathway on level 2 of the KEGG functional category (Fig. 1C). Circadian rhythm is one of the top 20 affected pathways in the environmental adaptation implicated by DEGs (Fig. 1B). The larger the Rich factor, the greater the enrichment. Circadian rhythm pathway showed the greatest enrichment (Fig. 1B). In KEGG level 3, 294 KEGG pathways including the circadian rhythm pathway between the two groups changed significantly (Supplement Data). A gene that draws our attention was that encoding for the mouse *Per1*, the core clock gene in negative-feedback loops (Fig. 1C, D). Circadian rhythm pathway contained 10 changed genes (Fig. 1D), eight genes were upregulated and two gene was downregulated (Fig. 1D). However, other core clock genes including *Clock, Bmal1, Cry1, Cry2*, and *Per2*, were no obvious changes, and only *Per1* was specifically elevated in livers following CCl_4_ exposure.

### Response changes of clock genes in mice exposed to CCl_4_

**Fig. 2:**
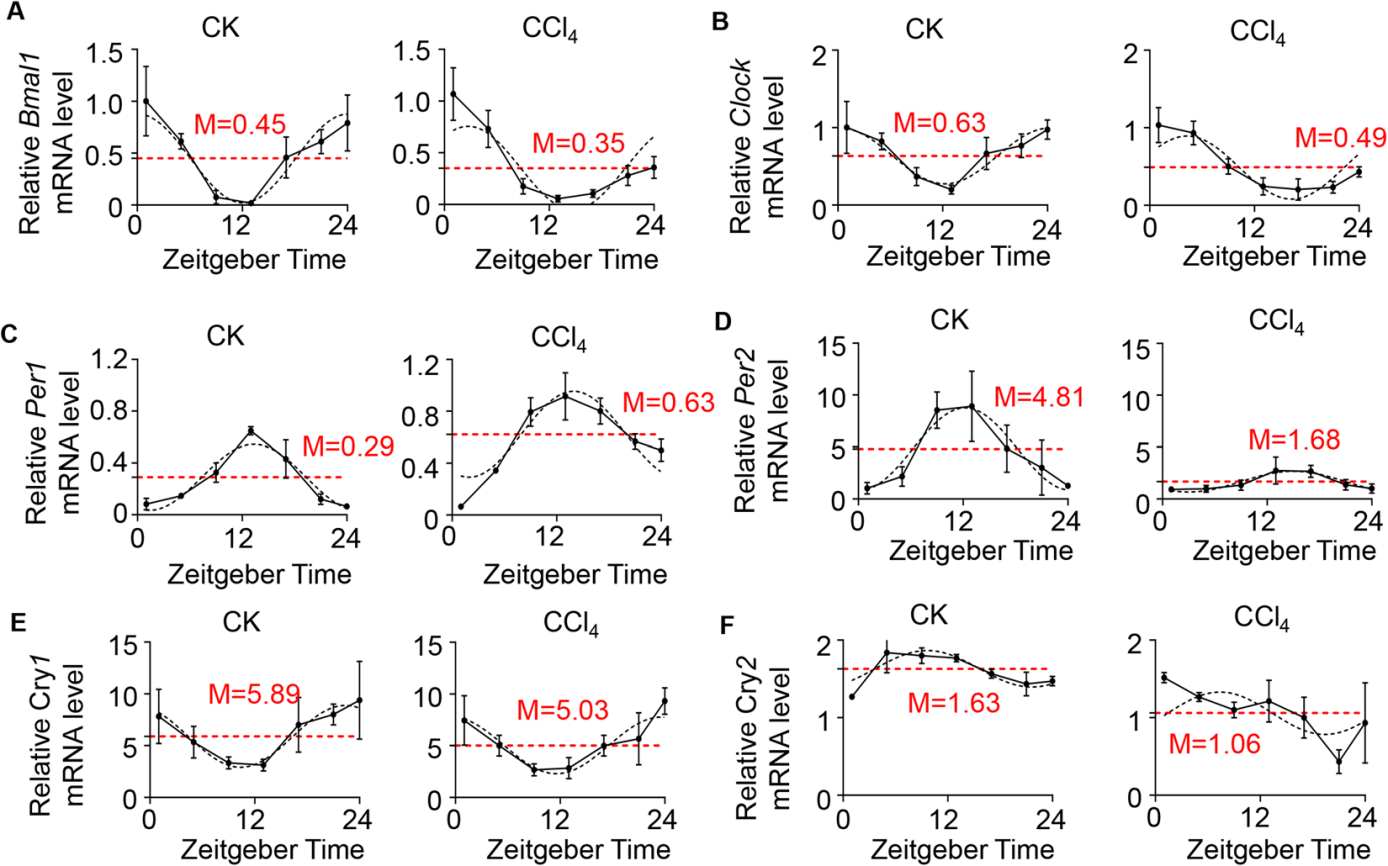
Circadian expression of clock genes in control and CCl_4_-treated mice. A single intraperitoneal injection of CCl_4_ (0.6 mL/kg body weight) was administrated to WT at ZT1. Mice were killed at ZT1, ZT5, ZT9, ZT13, ZT17, ZT21, and ZT24. Hepatic mRNA levels of *Bmal1* **A**, *Clock* **B**, *Per1* **C**, *Per2* **D**, *Cry1* **E**, and *Cry2* **F** were measured by real-time PCR after CCl_4_ treatment. CK: control. Solid lines indicate experimental response curves; dashed lines, fitted model curves. Red dashed line with the number represents mesor. Data were shown as means ± S.E.M. *N* = 5 independent biological replicates per group. Data represent cumulative results from three independent experiments.

To validate the results obtained from RNA-Seq, we carried out real-time PCR analysis for core clock genes expression in the liver mRNA between CCl_4_ and control group. As shown in Fig. 2, in both experimental and control groups, the core clock gene expressions were highly significantly changed within 24 hours. The present results confirmed that CCl_4_ acutely increased the expression of *Per1* in livers (Fig. 2C). Table 1 showed the mesor of *Per1* was significantly higher in CCl_4_-treated mice. While *Per1* mRNA levels dramatically elevated, the mRNA rhythmicities of other clock genes were maintained and significantly declined in CCl_4_-treated mice livers (Fig. 2A–F). These findings revealed that CCl_4_-induced liver injury resulted in changes in the expression of circadian clock genes with a specific elevation of *Per1* mRNA. Therefore, these observations raised a fundamental question to the biological relevance for such gene regulations.

**Table 1:** Circadian rhythmic parameters of clock gene transcriptions in control and CCl_4_-treated mice.

Gene	Mesor	Amplitude	Acrophase ZT (h)
Control			
*Bmal1*	0.45 ± 0.09	0.43 ± 0.12	0.08 ± 1.22
*Clock*	0.63 ± 0.08	0.36 ± 0.11	0.27 ± 1.33
*Per1*	0.29 ± 0.04	0.25 ± 0.05	13.33 ± 0.88
*Per2*	4.81 ± 0.93	3.95 ± 1.24	12.23 ± 1.34
*Cry1*	5.89 ± 0.92	2.94 ± 1.24	22.36 ± 1.83
*Cry2*	1.63 ± 0.08	0.23 ± 0.10	9.63 ± 1.85
CCl_4_			
*Bmal1*	0.35 ± 0.11	0.40 ± 0.15	2.71 ± 1.49
*Clock*	0.49 ± 0.10	0.41 ± 0.15	4.28 ± 1.22
*Per1*	0.63 ± 0.08**	0.33 ± 0.10	13.95 ± 1.15
*Per2*	1.68 ± 0.29*	1.00 ± 0.40	15.10 ± 1.49
*Cry1*	5.03 ± 0.78	2.73 ± 1.02	23.58 ± 1.64
*Cry2*	1.06 ± 0.17*	0.27 ± 0.24	7.38 ± 4.28

Data represent means ± S.E.M., *P < 0.05, **P < 0.01 vs. control group.

### *Per1* deficiency decreased the metabolism rate of toxins

**Fig. 3:**
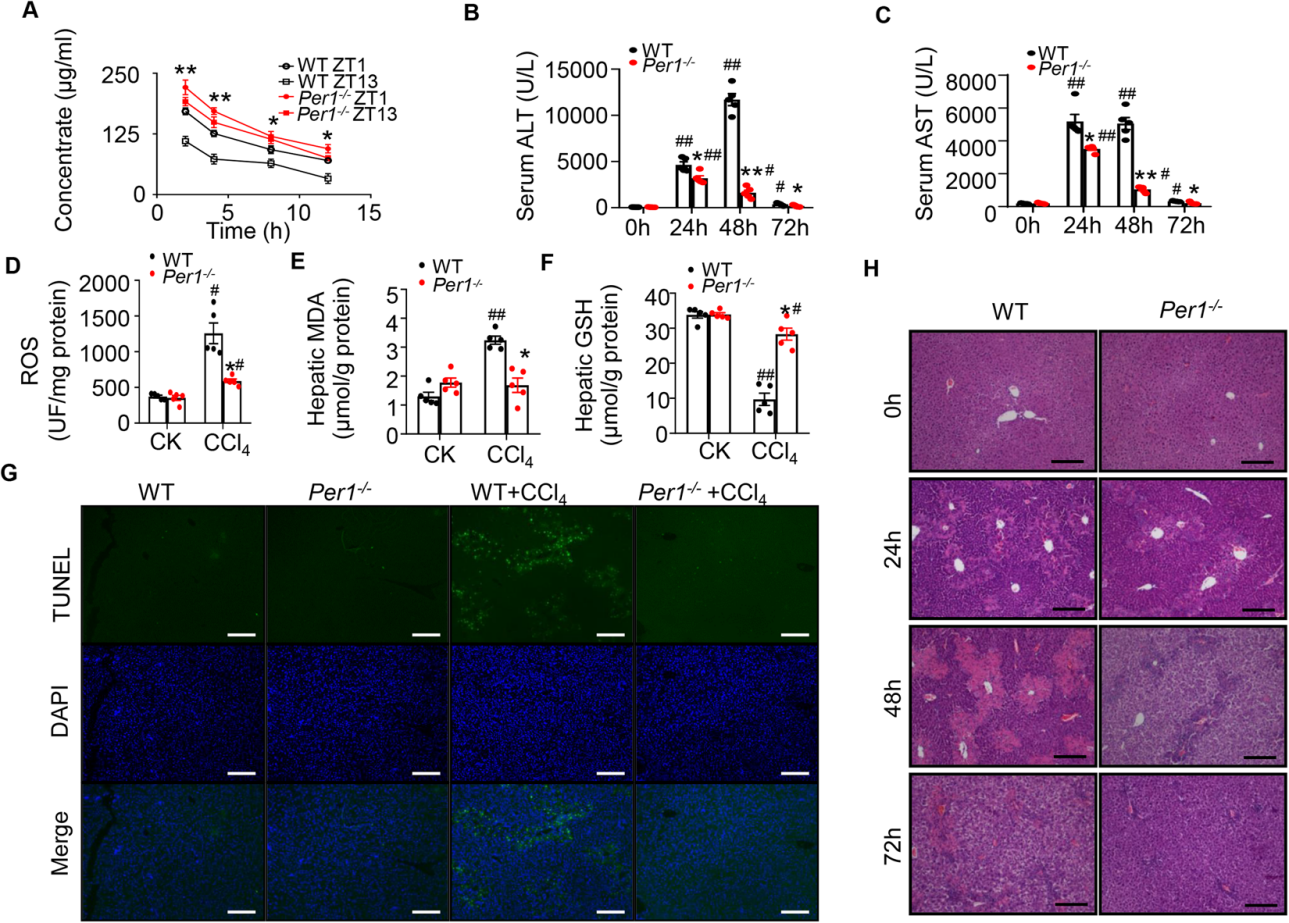
Effect of Per1 deficiency on CCl4-induced acute liver injury in mice. **A** The time course of plasma CCI_4_ concentrations from 0 to 12 h following a single intraperitoneal injection of CCl_4_ (0.6 mL/kg body weight). **B** Serum activities of ALT and **C** AST at 24, 48, and 72 h after single CCl_4_ treatment were measured. Liver tissues were collected 24, 48, and 72 h after single CCl_4_ treatment. Hepatic concentrations of **D** ROS production, **E** MDA, and **F** GSH were measured 24 h after single CCl_4_ treatment. **G** Representative immunostaining images. Apoptotic cells were visualized by TUNEL staining (green) and counterstained with DAPI (blue). **H** Representative H&E staining of livers from each treatment group. CK: control. Bar = 100 μm. Representative images from N = 3 biological replicates. Data were shown as means ± S.E.M. *N* = 5 independent biological replicates per group. * indicates *P* value < 0.05, ** indicates *P* value < 0.01, *Per1*^*−/−*^ group versus WT group; ^#^ indicates *P* value < 0.05, ^##^ indicates *P* value < 0.01, CCl_4_ group versus control group. Data represent cumulative results from three independent experiments.

In order to show that the specific expression of *Per1* caused by toxins is related to toxin degradation, a biochemical assay was undertaken to measure CCl_4_ residual levels in the plasma of WT and *Per1* knockout (*Per1*^−/−^) mice after given a single intraperitoneal injection of CCl_4_. Plasma extract obtained from CCl_4_-treated *Per1*^−/−^ mice displayed a higher level of CCl_4_ residue compared with that of WT mice, indicating loss of *Per1* decreased liver metabolism rate of CCl_4_ (Fig. 3a). Next, we investigated whether the metabolism of other toxins was slowed down in *Per1*^−/−^ mice. Certain concentrations capsaicin and APAP were intragastrically or intraperitoneally administrated into two genotypes of mice, respectively. The results showed that the residues of capsaicin in the plasma of *Per1*^−/−^ mice were significantly higher than those in wild-type mice, either in the morning or at night (Fig. S1A). Moreover, more APAP residues in plasma were observed after the APAP intraperitoneal administration in *Per1*^−/−^ mouse (Fig. S1B). Together, these results indicated that *Per1* deficiency reduced the metabolic rate of toxins.

### *Per1* deficiency decreased CCl_4_-induced acute liver injury

Then, we found an alleviated liver injury in *Per1*^−/−^ mice, reflecting with significantly decreased AST and ALT levels at 24, 48, and 72 h after CCl_4_ administration (Fig. 3B, C). The levels of hepatic ROS were also significantly lower in *Per1*^−/−^ mice compared with WT mice (Fig. 3D), but the MDA levels did not significantly change after CCl_4_ (Fig. 3E). The hepatic GSH level was reduced by CCl_4_ administration, with a further reduction in WT mice compared with *Per1*^−/−^ mice (Fig. 3F). Apoptotic cells were visualized by TUNEL staining and counterstained with DAPI. Treatment with CCl_4_ (24 h) induced a significant apoptotic response, consistent with the above results (Fig. 3G). Apoptosis was significantly less frequent in the liver of *Per1*^−/−^ mice as compared with WT mice, as seen by TUNEL (Fig. 3G). Histological examination of the liver tissues revealed that hepatic damage was limited to pericentral areas in mice at 24 h (Fig. 3H). The necrosis was less severe in the *Per1*^−/−^ mice compared with WT mice during 24–72 h after CCl_4_ administration. These observations suggested that the elevation of *Per1* expression in toxin CCl_4_ exposure was associated with the enhancement of liver metabolism of toxins capability.

### *Per1* deficiency alleviated chronic CCl_4_-induced liver injury and fibrosis in mice

**Fig. 4:**
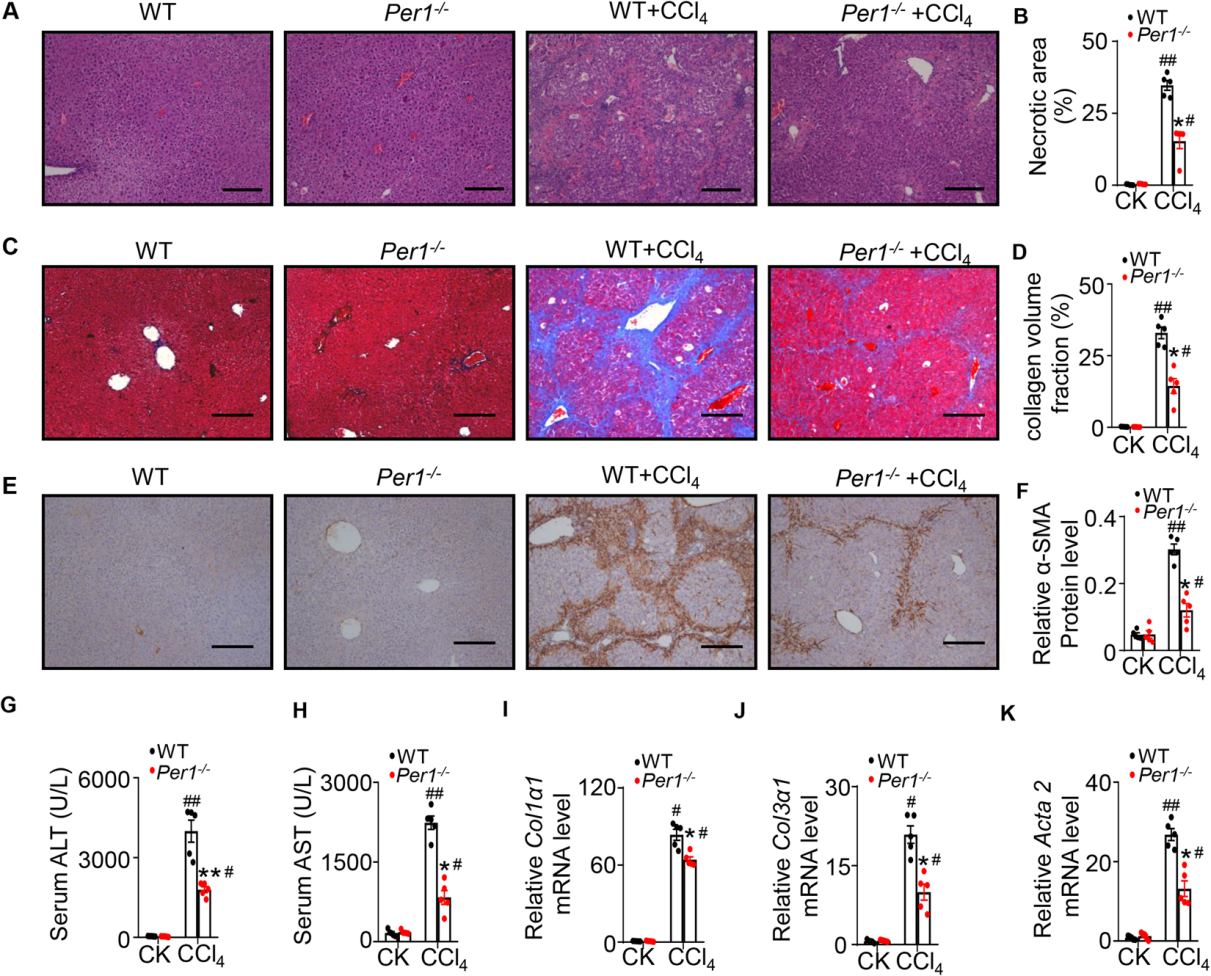
Effect of Per1 deficiency on liver injury induced by chronic CCl4 administration. **A** H&E staining of livers from each treatment group. **B** The percentage of necrotic area was quantified using Image J software. **C** Masson’s trichrome of livers from each treatment group for the visualization of hepatic collagen deposition. **D** The percentage of collagen volume fraction was quantified using Image J software. **E** Expression of *α*-SMA was determined by immunohistochemistry. **F** Relative α-SMA Protein level was quantified using Image J software. Bar = 100 μm. Representative images from *N* = 3 biological replicates. Serum activities of **G** ALT and **H** AST were measured after chronic CCl_4_ administration. Relative mRNA levels of **I** *Col1α1* and **J** *Col3α1* were measured in the liver of WT and *Per1*^−/−^ mice. **K** Relative mRNA levels of *Atca2* were measured in liver of WT and *Per1*^−/−^ mice. CK: control. Data were shown as means ± S.E.M. *N* = 5 independent biological replicates per group. * indicates *P* value <0.05, ** indicates *P* value <0.01, *Per1*^−/−^ group versus WT group; ^#^ indicates *P* value < 0.05, ^##^ indicates *P* value <0.01, CCl_4_ group versus control group.

To expand our observation that loss of *Per1* reduced CCl_4_-induced liver acute injury, we performed a series of experiments to assess CCl_4_-induced chronic liver injury and fibrosis in WT and *Per1*^−/−^ mice. Mice were injected with 0.6 mL/kg body weight CCl_4_ twice weekly for 4 weeks and killed 48 h after the last injection. As expected, histological examination of the liver tissues after CCl_4_ administration also revealed significantly less severe necrosis in *Per1*^−/−^ mice compared with WT mice (Fig. 4A, B). Chronic liver injury was alleviated in *Per1*^−/−^ mice as evidenced by lower serum ALT and AST levels after CCl_4_ administration (Fig. 4G, H). After chronic treatment of CCl_4_ for 4 weeks, WT mice had significant hepatic fibrosis, as demonstrated by Masson’s trichrome staining. In contrast, *Per1*^−/−^ mice had less fibrosis as demonstrated by reduced collagen deposition (Fig. 4C, D). Expression of α-SMA, a marker of hepatic stellate cell (HSC) activation, was increased in both genotypes in response to chronic CCl_4_ treatment. *Per1* deficiency significantly reduced α-SMA expression after CCl_4_ administration as assessed by immunohistochemistry (Fig. 4E, F). CCl_4_ administration elevated mRNA levels of markers for fibrogenesis including *Col1α1*, *Col3α1*, and *Acta2*. The expression of all these genes after CCl_4_ treatment was significantly suppressed in *Per1*^−/−^ mice compared with WT mice (Fig. 4I–K). These results indicated that *Per1* deficiency alleviated chronic CCl_4_-induced liver injury and fibrosis in mice.

### *Per1* deficiency declined the level of *Cyp2e1* expression in vivo

**Fig. 5:**
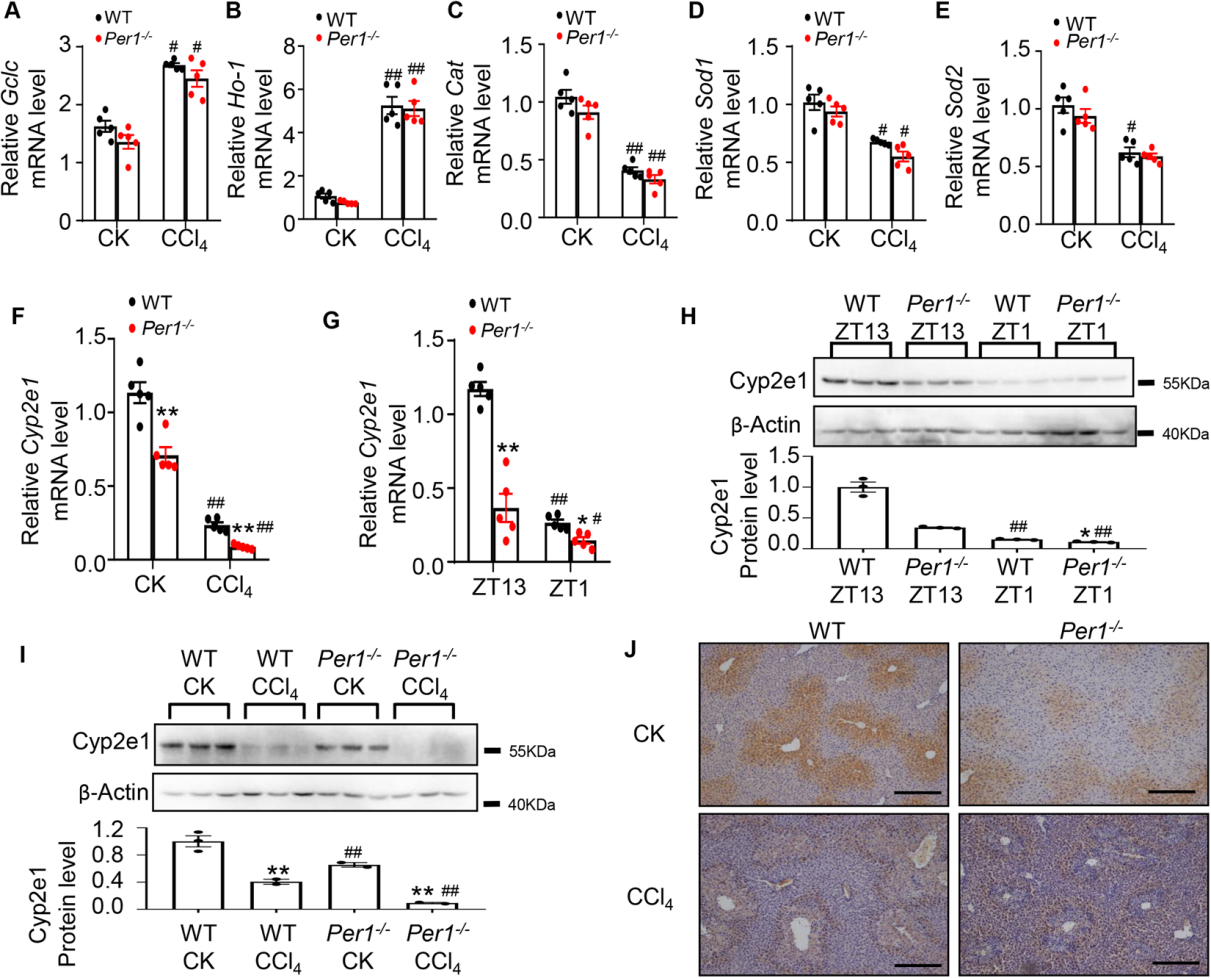
Effect of Per1 deficiency on Cyp2e1 expression exposed to CCl4. Liver tissues were collected 24 h after single CCl_4_ administration. Hepatic mRNA levels of **A** *Gclc*, **B** *Ho-1*, **C** *Cat*, **D** *Sod1*, **E** *Sod2*, and **F** *Cyp2e1* were measured by real-time PCR. CK: control. Data were shown as means ± S.E.M. *N* = 5 independent biological replicates per group. * indicates *P* value < 0.05, ** indicates *P* value <0.01, *Per1*^*−/−*^group versus WT group; ^#^ indicates *P* value <0.05, ^##^ indicates *P* value <0.01, CCl_4_ group versus control group. **G** *Cyp2e1* expression was measured by real-time PCR in the mouse liver at ZT1 and ZT13. **H** Representative western blots for rhythm of CYP2E1 protein expression in the mouse liver. Extracts were measured via western blot analysis with anti-CYP2E1 or anti–β-ACTIN antibody. CK: control. Data were shown as means ± S.E.M. *N* = 3 independent biological replicates per group. * indicates *P* value <0.05, ** indicates *P* value <0.01, *Per1*^*−/−*^ group versus WT group; ^#^ indicates *P* value <0.05, ^##^ indicates *P* value <0.01, ZT1 group versus ZT13 group. **I** Western blot assay of CYP2E1 protein after CCl_4_ treatment in vivo. **J** Representative images of immunohistochemical staining were shown for liver sections stained with anti-CYP2E1 antibody. This is in agreement with the RT-PCR and western blot data. Bar = 100 μm. Representative images from *N* = 3 biological replicates. CK: control. Data were shown as means ± S.E.M. *N* = 3 independent biological replicates per group. * indicates *P* value <0.05, ** indicates *P* value <0.01, *Per1*^*−/−*^ group versus WT group; ^#^ indicates *P* value <0.05, ^##^ indicates *P* value <0.01, CCl_4_ group versus control group. Data represent cumulative results from three independent experiments.

As circadian genes can influence hepatic oxidative stress by mediating the expression of antioxidative enzymes in mice liver^27,28^, we examined the effect of *Per1* deletion on hepatic expression of these enzymes, including *Gclc*, *Ho-1*, *Cat*, *Sod1*, and *Sod2*. CCl_4_ treatment significantly elevated the expression of *Gclc* and *Ho-1*. *Cat*, *Sod1*, and *Sod2* were downregulated in response to CCl_4_. These antioxidative enzymes showed no significant changes between WT and *Per1*^−/−^ mice (Fig. 5A–E). The injurious effects of a few toxins are results of their intermediary metabolites by CYP2E1, some highly toxic molecules that can lead to oxidative injury^29,30^. CCl_4_ is metabolized by CYP2E1 to trichloromethyl peroxyl radical that induces oxidative stress cell damage and cell death^31^. The change of *Cyp2e1* expression by qRT-PCR analysis was in agreement with RNA-seq data (Fig. S2). Intraperitoneal injection of mice with CCl_4_ decreased *Cyp2e1* expression as previously described^31^. Analysis of mRNA levels in the liver revealed that *Cyp2e1* expression was significantly downregulated by *Per1* deletion in both control and CCl_4_ group (Fig. 5F), indicating a possibility of decreased hepatic injury after CCl_4_ was primarily owing to downregulation of *Cyp2e1* in *Per1*^−/−^ mice. Then we investigated whether the expression of *Cyp2e1* was circadian controlled. The results showed the mRNA expression of *Cyp2e1* was maximal at the beginning of the dark phase of the entraining photocycle at ZT13 and minimal at ZT1, the onset of the light phase (Fig. 5G). The knockdown of *Per1* resulted in an approximately twofold diminution of *Cyp2e1* expression at the mRNA level (Fig. 5G). Western blot analysis revealed that CYP2E1 protein levels were higher during the dark phase (Fig. 5H). The CYP2E1 protein was also reduced in *Per1*^−/−^ mice, and the circadian rhythm of CYP2E1 was impaired (Fig. 5H). Analysis of protein levels in the liver revealed that CYP2E1 expression was significantly downregulated by *Per1* deletion in both control and CCl_4_ group (Fig. 5I). Next, we performed immunoblotting analysis to examine putative changes in CYP2E1 protein levels in mouse liver tissues, resulting from CCl_4_ treatment. Parallel immunohistochemical examination revealed that CYP2E1-positive staining was decreased in *Per1*^−/−^ mice exposed CCl_4_ compared with WT mice (Fig. 5J). Taken together, these findings demonstrated that *Per1* participated in the regulation of transcriptional activation of *Cyp2e1* and, in turn, played a critical role in mediating CCl_4_-induced hepatotoxicity.

### Effect of *Per1* on CCl_4_-induced oxidative injury and *Cyp2e1* expression in hepatocytes

**Fig. 6:**
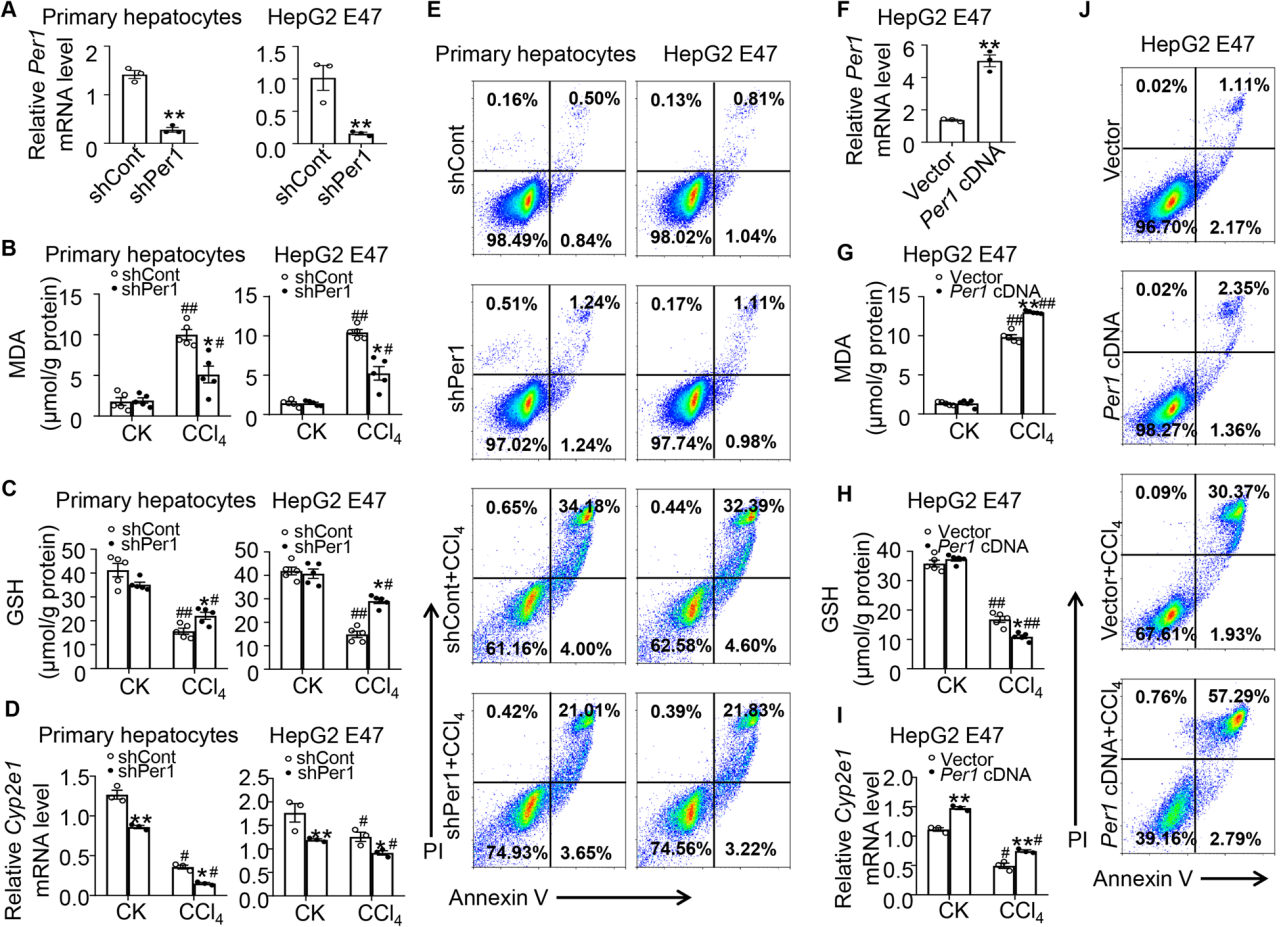
Per1 mediated the regulation of Cyp2e1 expression in vitro. **A** Knockdown of *Per1* was verified by real-time PCR after infection with shPer1 or shRNA control (shCont) in primary cells and HepG2 E47 cells. Cells were harvested after treatment with 0.5% CCl_4_ for 24 h. Levels of **B** MDA and **C** GSH in cell lysates were determined. **D** *Cyp2e1* expression was measured by real-time PCR. **E** Representative images of flow cytometry analysis of apoptosis by annexin V/PI staining in primary cells and HepG2 E47 cells infected with shPer1 or shCont. CK: control. Data were shown as means ± S.E.M. *N* = 3–5 independent biological replicates per group. * indicates *P* value <0.05, ** indicates *P* value <0.01, shPer1 group versus shCont group; ^#^ indicates *P* value <0.05, ^##^ indicates *P* value < 0.01, CCl_4_ group versus control group. HepG2 cells were transfected with *Per1* cDNA or vector as control. **F** Real-time PCR was used to analyze changes in gene expression of *Per1*. Levels of **G** MDA and **H** GSH in cell lysates were determined. **I** *Cyp2e1* expression was measured by real-time PCR. **J** Representative flow profile of annexin V/PI staining. CK: control. Data were shown as means ± S.E.M. *N* = 3–5 independent biological replicates per group. * indicates *P* value <0.05, ** indicates *P* value <0.01, *Per1* cDNA group versus vector group; ^#^ indicates *P* value <0.05, ^##^ indicates *P* value <0.01, CCl_4_ group versus control group. Data represent cumulative results from three independent experiments.

To investigate whether *Per1* directly regulated *Cyp2e1* expression in hepatocytes, we analyzed CCl_4_-induced oxidative injury in primary hepatocytes and HepG2 E47 cells. The primary hepatocytes were transfected with control shRNA or *Per1*-specific shRNA, and the cells were challenged with CCl_4_. shRNA-mediated knockdown of *Per1* led to a 70–80% decrease in mRNA levels (Fig. 6A). Next, we created stable HepG2 E47 cell lines expressing shPer1 through retroviral infection and assessed the effect of *Per1* suppression on the expression of *Cyp2e1*. As expected, real-time PCR experiments showed that cells treated with *Per1* shRNA decreased the expression level of *Per1* (Fig. 6A). CCl_4_ exposure increased intracellular MDA levels, and suppression of *Per1* expression significantly impaired the elevation of MDA in primary hepatocytes (Fig. 6B). The contents of GSH in cells also decreased after CCl_4_, which could be alleviated by knockdown of *Per1* in primary hepatocytes (Fig. 6C). *Per1* inhibition improved the increase in MDA levels and the decrease in GSH levels in HepG2 E47 cells (Fig. 6B, C). *Per1* inhibition also effectively reduced mRNA levels of *Cyp2e1* in primary hepatocytes (Fig. 6D). *Per1* inhibition reduced *Cyp2e1* mRNA levels in HepG2 E47 cells, consistent with the results of primary hepatocytes (Fig. 6D). Apoptosis was measured by annexin V/PI staining followed by flow cytometry. Compared with control shRNA, the cells treated with *Per1* shRNA showed less apoptosis in vitro in primary cells (Fig. 6E). Consistent with the above observation, inhibition of *Per1* expression resulted in the reduction of apoptosis in HepG2 E47 cells (Fig. 6E). To further study the function of *Per1*, HepG2 E47 cells expressing *Cyp2e1* were transfected with *Per1-*cDNA plasmid and then treated with CCl_4_ for 24 h. Overexpression was verified by real-time PCR (Fig. 6F). Overexpression of *Per1* in HepG2 E47 increased CCl_4_ toxicity. Moreover, MDA induced by CCl_4_ was increased to a greater extent, and GSH was further decreased (Fig. 6G, H).The *Per1* transfection significantly increased *Cyp2e1* expression in hepatocytes both in control and CCl_4_ group (Fig. 6I), which was consistent with that observed in vivo. We observed a significant increase in apoptosis after CCl_4_ in cells of *Per1* cDNA transfected (Fig. 6J).

### The *Per1* increased *Cyp2e1* expression by interacting with HNF-1α on *Cyp2e1* promoter

**Fig. 7:**
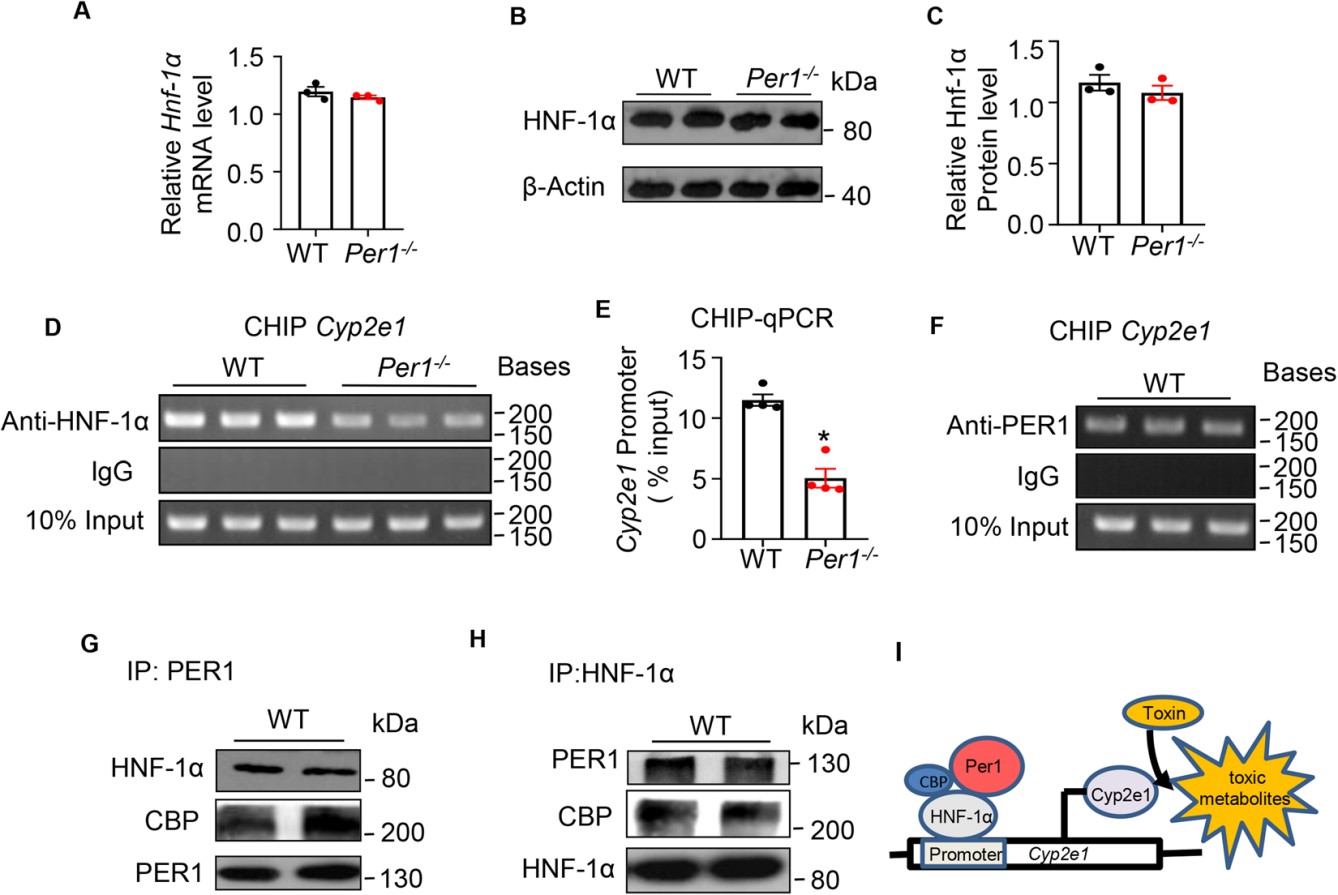
PER1 interacted with HNF-1α on Cyp2e1 promoter. **A** Hepatic mRNA levels of *HNF-1α* were measured in *Per1*^−/−^ and WT mice. **B**–**C** Hepatic protein levels of HNF-1*α* were analyzed by western blot and β-actin was used as internal control. **D** Recruitments of HNF-1*α* to *Cyp2e1* promoter were detected by ChIP assays. **E** ChIP assays were performed followed by real-time PCR. **F** Recruitments of PER1 to *Cyp2e1* promoters were detected by ChIP assays. **G**–**H** Representative immunoblots showing recovery of the indicated proteins from mouse liver after immunoprecipitation with the indicated antibodies. **I** A hypothetical model of how PER1 interacted with HNF-1α on *Cyp2e1* promoter and target gene transcription. Data were shown as means ± S.E.M. *N* = 5 independent biological replicates per group; ^*^P < 0.05, *Per1*^−/−^ group versus WT group.

Next, we investigated how *Per1* regulates *Cyp2e1* expression. *Cyp2e1* exhibits 24-hour periodicity in its hepatic mRNA levels, and this circadian rhythm is regulated by HNF-1α and the circadian organization of molecular clocks^12^. Both hepatic mRNA and protein levels of HNF-1α were similar between WT and *Per1*^−/−^ mice (Fig. 7A–C). The role of PER1 in the transcriptional activation of HNF-1α was confirmed by ChIP-qPCR, which showed a marked reduction (about twofold) in HNF-1α binding to *Cyp2e1* promoter regions in *Per1*-deficient liver tissues (Fig. 7D, E). PER1 protein also occurred in the HNF-1α-binding site in the *Cyp2e1* promoter (Fig. 7F), implying a possible interaction between PER1 and HNF-1α. Co-immunoprecipitation and western blot analysis of the liver lysates showed that HNF-1α interacted with PER1 and forms a protein complex (Fig. 7G). Interaction of CREB binding protein (CBP) with the N-terminal domain of HNF-1 greatly increased the binding affinity^32^. To further verify the specificity of HNF-1α to the PER1, we tested the binding of CBP to PER1. CBP was also co-immunoprecipitated by the anti-PER1 (Fig. 7G). Not surprisingly, we also observed strongly synergy between PER1 and HNF-1α, whereas CBP specifically interacted with HNF-1α in the whole-liver homogenates immunoprecipitated with anti-HNF-1α antibody (Fig. 7H). These results indicated that PER1 protein interacted with the HNF-1α–CBP complex and increased the binding activity of HNF-1α on the *Cyp2e1* promoter, enhancing the transcription of *Cyp2e1* in mice liver.

## Discussion

Our observations that toxins specifically activate *Per1* expression illustrate the central role played by the circadian clock in mediating the metabolism of toxins physiology in mice. Our previous study showed that *Per2* plays a protective role in CCl_4_-induced hepatotoxicity^33^. The opposite gene expression of *Per1* and *Per2* in mice exposed to CCl_4_ suggested different roles of core circadian genes. *Per2* gene functions in hepatocyte protection from chemical toxicants via the regulation of hepatic *Ucp2* gene expression levels^33^. Loss of *Per1* reduced metabolism of toxins capability, and eliminated the difference of toxin residues in plasma between day and night, suggesting *Per1* is required for maintaining daily xenobiotic-metabolizing rhythms. It is well known that *Cyp2e1* is responsible for the metabolism of most xenobiotics and is responsible for the efficient elimination of foreign chemicals from the body^34^. Our results supported that the metabolizing capacity of *Per1* is through regulating *Cyp2e1* expression. Paradoxically, *Cyp2e1* metabolically activates biologically inert compounds to electrophilic derivatives that can cause toxicity and cell death^10,11^. CYP2E1 catalyzes CCl_4_ molecule to the trichloromethyl free radical and induces hepatotoxicity. Elevation of CYP2E1 activity with phenobarbital, dichlorodiphenyltrichloroethane, or Arochlor 1254 R increases the hepatotoxic effect of CCl_4_^35^. Mice lacking *Cyp2e1* are resistant to CCl_4_ hepatotoxicity^36^. Therefore, it is reasonable that mice lacking *Per1* alleviate CCl_4_-induced liver injury and fibrosis due to the low level of hepatic *Cyp2e1*.

The *Cyp2e1* expression and activities are circadian controlled with higher levels in the dark phase in mice liver^12,37^, and this rhythm was impaired in *Per1*^*−/−*^ mice. Previous study has demonstrated transcription of the *Cyp2e1* is rhythmically enhanced by HNF-1α, and circadian gene CRY1 rhythmically inhibits the binding activity of HNF-1α on *Cyp2e1* promoter^12,38,39^. PER and CRY play a central role in negative-feedback loop in the core clock system. The circadian pattern of *Per1* mRNA in the liver is consistent with the rhythmic expression of *Cyp2e1*, which peaks at ZT12 as previously described^40^. These evidences implied a possible role of *Per1* in regulating the circadian transcription of *Cyp2e1*. In the present study, we showed that *Per1* directly enhanced mRNA levels of *Cyp2e1*. The expression of HNF-1α was not affected by *Per1*. However, the recruitment of HNF-1α to *Cyp2e1* promoter was significantly weakened in *Per1*^−/−^ mice. ChIP assays also revealed the recruitment of PER1 protein to the binding site of HNF-1α in the *Cyp2e1* promoter. Previous study of the crystal structure of PER1 protein demonstrated that PER1 cannot directly bind to DNA^41^, implying that PER1 may associate with other proteins and indirectly bind to the *Cyp2e1* promoter. Results of co-immunoprecipitated assays revealed a novel interaction between PER1 and HNF-1α. The present study suggests that the *Per1* maintains the daily metabolism of toxin rhythm through regulation of *Cyp2e1* expression (Fig. 7I). Our findings presented a control system that causes alternating turn-off/turn-on of transcription on *Cyp2e1* by endogenous circadian PER1 interaction.

The overarching evidences have already showed a reciprocal regulation of circadian rhythms and metabolic states. Disrupted rhythms can lead to metabolic impairments, the reverse is also true that disrupting metabolism can alter circadian rhythms^3^. Patients with cirrhosis have liver portal hypertension and dysfunctional circadian clock systems^5^. Hepatic fibrosis induced by CCl_4_ leads to alterations in the rhythms of hepatic clock genes^6^, and the patients with hepatic fibrosis suffer abnormal sleeping cycles^42^, indicating possible side-effects of clock responding to liver injury and metabolic diseases. *Per1* is the unique clock gene that responds rapidly to toxin exposure, and this response inevitably influences the normal circadian system.

There are multiple CRE binding sites in the promoter of *Per1* gene, which may be caused by cAMP signal (enriched in KEGG). Loss of *Per1* reduced significantly CCl_4_-induced acute and chronic liver injury. Thus, appropriate suppression of *Per1* response is a potential therapeutic target for toxin-induced hepatotoxicity, also is a promising method to improve the dysfunctional circadian clock system caused by toxin metabolism.

## Supplementary information

Supplementary Figure legend

Table S1

Figure S1

Figure S2

Supplentment Data

## Data Availability

Data are available upon request from the corresponding author.
