# Supplementary material for: Period1 mediates rhythmic metabolism of toxins by interacting with CYP2E1
Source: Cell Death Dis. 2021 Jan 12;12(1):76. doi: 10.1038/s41419-020-03343-7 (PMC7804260; doi:10.1038/s41419-020-03343-7)
Supplement: Supplementary file 1 — Supplementary Figure legend [file 41419_2020_3343_MOESM1_ESM.doc]

Figure S1. *Period1* mediates rhythmic metabolism of toxins. (A) The time course of plasma capsaicin concentrations from 0 to 3 h following capsaicin treatment. WT and *Per1*^-/-^ mice were given capsaicin at a dose of 300 mg/kg, via gavage. (B) The time course of plasma capsaicin concentrations from 0 to 4 h following the intraperitoneal administration of 500 mg/kg of acetaminophen (APAP). Data were shown as means ± S.E.M of n = 5 animals per group. * indicates *P* value < 0.05, ** indicates *P* value < 0.01, *Per1^-/-^* group versus WT group.

Figure S2. *Cyp2e1* mRNA levels were decreased significantly in mice exposed to carbon tetrachloride identified by RNA-Seq analysis. RNA-seq–based gene expression values (FPKM) for *Cyp2e1* in CK and CCl_4_ group. CK: control. Asterisks indicate statistically significant differences (*q* < 0.0005) for expression in CCl_4_ (n = 3) relative to expression values in CK group.
