# Supplementary material for: Period1 mediates rhythmic metabolism of toxins by interacting with CYP2E1
Source: Cell Death Dis. 2021 Jan 12;12(1):76. doi: 10.1038/s41419-020-03343-7 (PMC7804260; doi:10.1038/s41419-020-03343-7)
Supplement: Supplementary file 2 — Table S1 [file 41419_2020_3343_MOESM2_ESM.docx]

**Table S1.** Primer sequences used for real-time RT-PCR

| *Gene* | *Forward/Reverse* | *Primer (5’ to 3’)* |
| --- | --- | --- |
| Mouse |  |  |
| *Gapdh* | Forward | CATCCACTGGTGCTGCCAAGGCTGT |
|  | Reverse | ACAACCTGGTCCTCAGTGTAGCCCA |
| *Bmal1* | Forward  Reverse | ACATAGGACACCTCGCAGAA  AACCATCGACTTCGTAGCGT |
| *Clock* | Forward  Reverse | CGGCGAGAACTTGGCATT  AGGAGTTGGGCTGTGATCA |
| *Per1* | Forward  Reverse | TCCTCAACCGCTTCAGAGATC  CGGGAACGCTTTGCTTTAGA |
| *Per2* | Forward  Reverse | GTGAAGCAGGTGAAGGCTAAT  AAGCTTGTAAGGGGTGGTGTAG |
| *Cry1* | Forward  Reverse | TTGCCTGTTTCCTGACTCGT  GACAGCCACATCCAACTTCC |
| *Cry2* | Forward  Reverse | TCGGCTCAACATTGAACGAA  GGGCCACTGGATAGTGCTCT |
| *Cyp2e1* | Forward | TCCCTAAGTA TCCTCCGTGA |
|  | Reverse | GTAA TCGAAGCGTTTGTTGA |
| *Ho-1* | Forward | GAGCCTGAA TCGAGCAGAAC |
|  | Reverse | AGCCTTCTCTGGACACCTGA |
| *Sod1* | Forward | GAACCA TCCACTTCGAGCA |
|  | Reverse | TACTGA TGGACGTGGAACCC |
| *Sod2* | Forward | CACATTAACGCGCAGATCATG |
|  | Reverse | CCAGAGCCTCGTGGTACTTCTC |
| *Cat* | Forward | ACCAGGGCATCAAAAACTTG |
|  | Reverse | GCCCTGAAGCTTTTTGTCAG |
| *Gclc* | Forward | GGGTGCTTGTTTATGGCTTC |
|  | Reverse | GTGGAGGCCAATATGAGGAA |
| *Hnf-1α* | Forward | CTGAAAGAGCCGGAGAACCT |
|  | Reverse | GACTTGACCATCTTCGCCAC |
| *Acta2* | Forward | ATGAAGCCCAGAGCAAGAGA |
|  | Reverse | ATGTCGTCCCAGTTGGTGAT |
| *Col1α1* | Forward | ACGTCCTGGTGAAGTTGGTC |
|  | Reverse | CAGGGAAGCCTCTTTCTCCT |
| *Col3α1* | Forward | TGGTCCTCAGGGTGTAAAGG |
|  | Reverse | GTCCAGCATCACCTTTTGGT |
| Human |  |  |
| *Gapdh* | Forward | CTCTGCTCCTCCTGTTCGAC |
|  | Reverse | TTAAAAGCAGCCCTGGTGAC |
| *Per1* | Forward | AGTCCGTCTTCTGCCGTATCA |
|  | Reverse | AGCTTCGTAACCCGAATGGAT |
| *Cyp2e1* | Forward | GACCACCAGCACAACTCTGA |
|  | Reverse | CCCAATCACCCTGTCAATTT |
| ChIP |  |  |
| *Cyp2e1* promoter | Forward | GTCAGATCAGTAGATGCAAC |
|  | Reverse | TAGGAATTTCCTGAGGAGAA |
