# Supplementary figures and images for: Period1 mediates rhythmic metabolism of toxins by interacting with CYP2E1
Source: Cell Death Dis. 2021 Jan 12;12(1):76. doi: 10.1038/s41419-020-03343-7 (PMC7804260; doi:10.1038/s41419-020-03343-7)

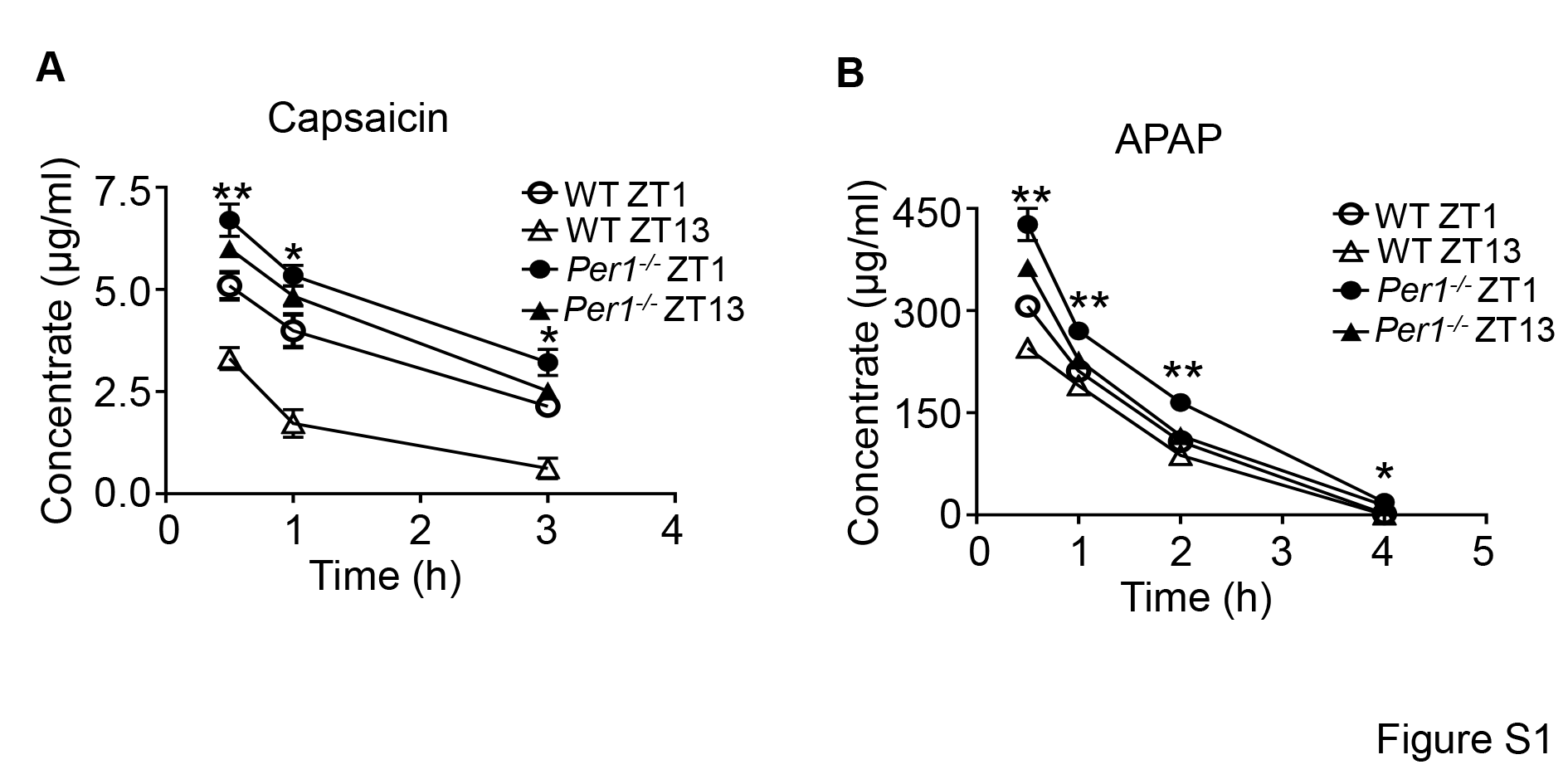

Supplement: Supplementary file 3 — Figure S1 [file 41419_2020_3343_MOESM3_ESM.png]

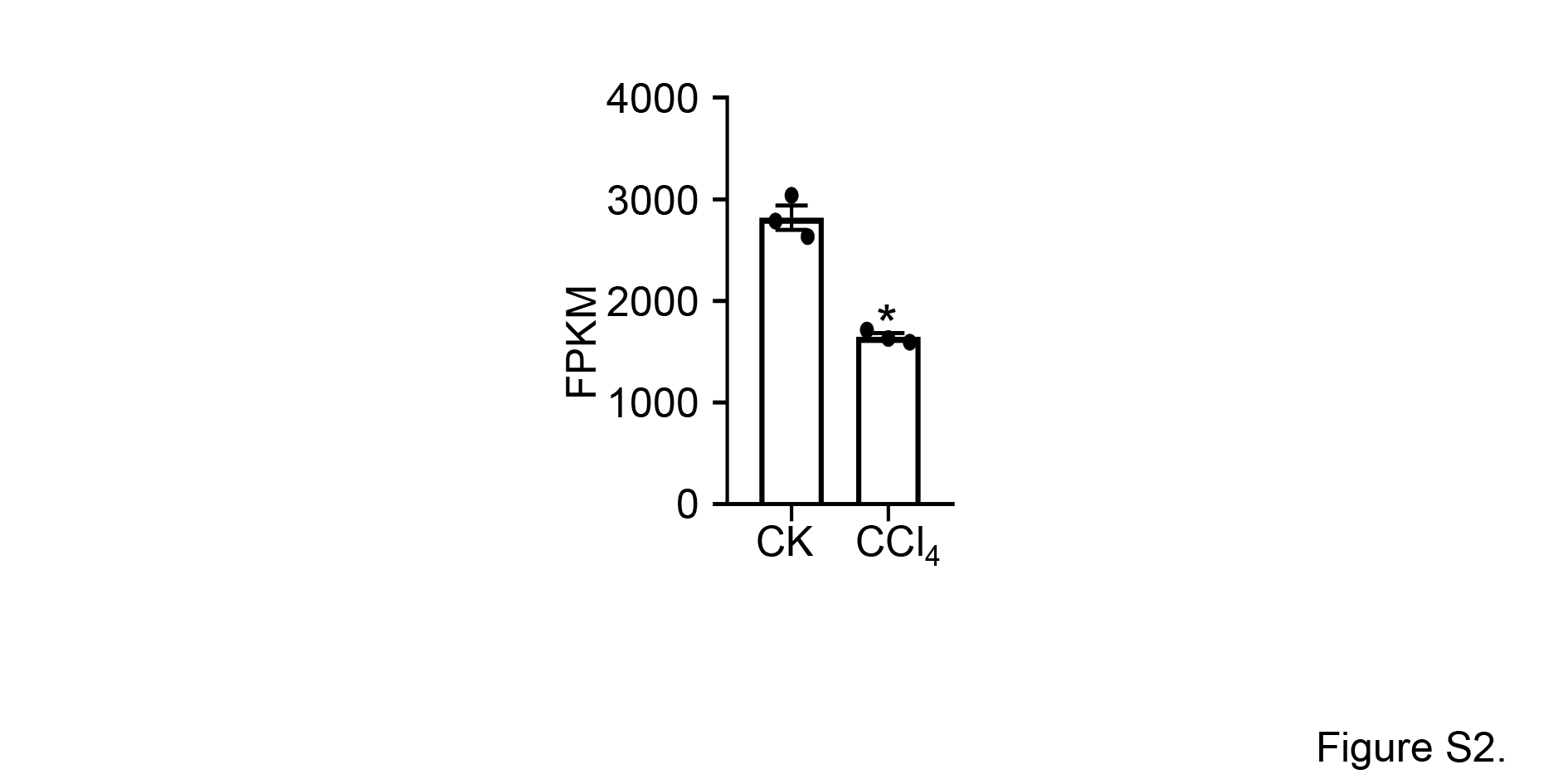

Supplement: Supplementary file 4 — Figure S2 [file 41419_2020_3343_MOESM4_ESM.png]
